# Effect of the Reversed L-Shaped Osteotomy on the Round Sign: Not All Hallux Valgus Deformities May Need Proximal Derotation to Correct the Radiographic Appearance of Metatarsal Pronation

**DOI:** 10.1177/24730114221115697

**Published:** 2022-08-05

**Authors:** Lizzy Weigelt, Linda Wild, Elin Winkler, Carlos Torrez, Thorsten Jentzsch, Stephan H. Wirth

**Affiliations:** 1 Department of Orthopedics, University Hospital Balgrist, University of Zurich, Zurich, Switzerland

**Keywords:** hallux valgus, metatarsal pronation, round sign, ReveL osteotomy

## Abstract

**Background:**

Metatarsal pronation has been claimed to be a risk factor for hallux valgus recurrence. A rounded shape of the lateral aspect of the first metatarsal head has been identified as a sign of persistent metatarsal pronation after hallux valgus correction. This study investigated the derotational effect of a reversed L-shaped (ReveL) osteotomy combined with a lateral release to correct metatarsal pronation. The primary hypothesis was that most cases showing a positive round sign are corrected by rebalancing the metatarsal-sesamoid complex. We further assumed that the inability to correct the round sign might be a risk factor for hallux valgus recurrence.

**Methods:**

We retrospectively evaluated 266 cases treated with a ReveL osteotomy for hallux valgus deformity. The radiologic measurements were performed on weightbearing foot radiographs preoperatively, at an early follow-up (median, 6.2 weeks), and the most recent follow-up (median, 13 months). Univariate and multivariate logistic regression analyses identified risk factors for hallux valgus recurrence (hallux valgus angle [HVA] ≥ 20 degrees).

**Results:**

A preoperative positive radiographic round sign was present in 40.2% of the cases, of which 58.9% turned negative after the ReveL osteotomy (*P* < .001). Hallux valgus recurred in 8.6%. Risk factors for recurrence were a preoperative HVA >30 degrees (odds ratio [OR] = 5.3, *P* < .001), metatarsus adductus (OR = 4.0, *P* = .004), preoperative positive round sign (OR = 3.3, *P* = .02), postoperative HVA >15 degrees (OR = 74.9; *P* < .001), and postoperative positive round sign (OR = 5.3, *P* = .008). Cases with a positive round sign at the most recent follow-up had a significantly higher recurrence rate than those with a negative round sign (22.7% vs 5.9%, *P* < .001).

**Conclusion:**

The ReveL osteotomy corrected a positive round sign in 58.9%, suggesting that not all hallux valgus deformities may need proximal derotation to negate the radiographic appearance of the round sign. A positive round sign was found to be an independent risk factor for hallux valgus recurrence. Further 3-dimensional analyses are necessary to better understand the effects and limitations of distal translational osteotomies to correct metatarsal pronation.

**Level of Evidence:**

Level IV, case series.

## Introduction

Postoperative recurrence is one of the most common complications after hallux valgus surgery. Depending on the surgical technique and the definition of recurrence, rates from 8% to 73% have been reported.^2,13,25^ Several radiologic factors increasing the risk of hallux valgus recurrence have been described, including a higher preoperative hallux valgus angle (HVA),^9,22^ an insufficiently corrected postoperative HVA and intermetatarsal angle (IMA),^22^ incongruency of the first metatarsophalangeal joint (MTPJ),^6,9^ an increased distal metatarsal articular angle (DMAA),^25^ incomplete reduction of the sesamoids,^20^ residual hallux valgus interphalangeus,^12^ metatarsus adductus, and flat foot deformity.^11^

Hallux valgus has been recently investigated as a 3-dimensional deformity, with metatarsal pronation being one of the key features.^14,21,27^ Metatarsal pronation resembles the rotation of the first metatarsal in the coronal plane, causing the plantar surface of the bone to face laterally. Kim et al^14^ showed that metatarsal pronation was present in 87% of the 166 examined hallux valgus cases, of which 26% showed abnormal metatarsal pronation without any sesamoid displacement. Okuda et al^21^ introduced the round sign, a round-shaped appearance of the lateral edge of the first metatarsal head, assessed on dorsoplantar weightbearing foot radiographs. They classified the lateral edge into 3 types (round, intermediate, and angular) and found the round shape (ie, positive round sign) to be a risk factor for hallux valgus recurrence. The authors suggested that the round shape results from increased metatarsal pronation.^21^ Yamaguchi et al^30^ later confirmed that a positive round sign is significantly correlated with increased pronation and inclination of the first metatarsal. Based on these findings, the concept of proximal derotational osteotomies of the first metatarsal and derotational first tarsometatarsal joint fusions was introduced, which allows both correction of large deformities in the coronal plane and reduction of metatarsal pronation. Case series with short-term results of these rotational techniques showed promising results with significant reduction of the round sign and low recurrence rates.^29,31^ However, proximal osteotomies show a considerable complication rate and require an increased period of immobilization.^17,31^

**Figure 1.**
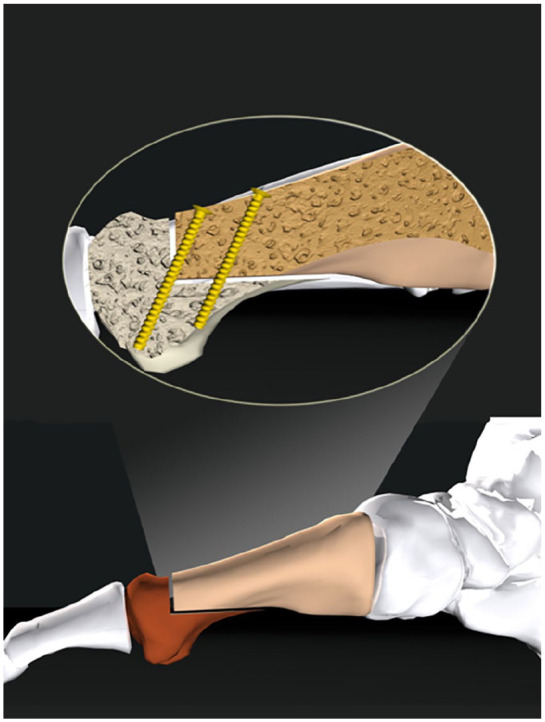
Illustration of the reversed L-shaped (ReveL) osteotomy.

The reversed L-shaped (ReveL) osteotomy is a modified chevron osteotomy with a short dorsal vertical limb and a longer plantar horizontal limb (Figure 1).^7^ This technique combines the advantages of the distal and the more proximal metatarsal osteotomies, namely, high corrective power and intrinsic mechanical stability, enabling early mobilization.^8^ By shifting the L-shaped distal metatarsal fragment laterally, this technique allows adequate deformity correction in the transverse and sagittal plane but has only limited effect on coronal rotation of the first metatarsal. However, in our practice, we often observed the round sign to turn negative with the ReveL osteotomy.

The objective of this retrospective study was to investigate the derotational effect of the ReveL osteotomy to correct metatarsal pronation. The primary hypothesis was that the ReveL osteotomy corrects metatarsal pronation by rebalancing the metatarsal-sesamoid joint complex, presented as a change in the round sign to negative in most cases. Second, patients with a persistent positive round sign were assumed to have a higher risk of hallux valgus recurrence.

## Methods

### Patient Selection

After obtaining ethics approval, the hospital database was screened for all consecutive patients who underwent ReveL osteotomy to correct hallux valgus deformity at our institution between January 2004 and December 2014 (548 feet, in the following referred to as cases). All cases with a preoperative hallux valgus angle (HVA) >15 degrees and a minimum 1-year radiologic follow-up were included in the study (362 cases). Exclusion criteria were missing preoperative or early postoperative (6 weeks to 3 months) radiographs (3 cases), previous surgery to the first ray (13 cases), other interventions to the first ray apart from an Akin osteotomy (eg, metatarsal-cuneiform joint fusion; 70 cases), and hallux rigidus requiring additional cheilectomy (10 cases).

### Surgical Technique

The skin incision was centered over the medial first MTPJ. During superficial dissection, the medial cutaneous branch of the superficial peroneal nerve was protected. The joint capsule was incised longitudinally and sharply released from the dorsal and medial metatarsal head, preserving the plantar soft tissues that contain the blood supply to the metatarsal head. The lateral joint capsule and metatarsal-sesamoid ligament were approached over the top of the metatarsal head and released under visual control from proximal to distal. The ReveL osteotomy was performed as previously described.^7^ The vertical cut of the osteotomy was set first and directed perpendicular to the second metatarsal shaft axis, which prevented shortening or lengthening of the first metatarsal. In cases with increased DMAA, an additional vertical cut to remove a medial-based wedge was performed for biplanar correction. The horizontal limb of the ReveL osteotomy was cut parallel to the sole of the foot, aiming toward the plantar cortex of the first metatarsal. The metatarsal head fragment was mobilized and shifted laterally up to 75% of its diameter, depending on the correction needed, and fixed with two 2.4-mm cortex screws in a dorsoplantar direction. The medial eminence was resected with an oscillating saw. During medial capsulorrhaphy, the hallux was held in a neutral position. Postoperatively, a specific dressing was applied to unload the medial capsule and maintain the corrected position of the hallux. Patients were instructed to fully weightbear through the heel in a postoperative shoe with a rigid sole for 6 weeks.

### Radiologic Evaluation

The radiologic measurements were performed on weightbearing anteroposterior and lateral foot radiographs, taken preoperatively, at an early follow-up, and at the most recent follow-up. The measurements were performed by 3 orthopaedic surgeons using a preoperative planning software (mediCAD; Hectec GmbH, Germany).

**Figure 2.**
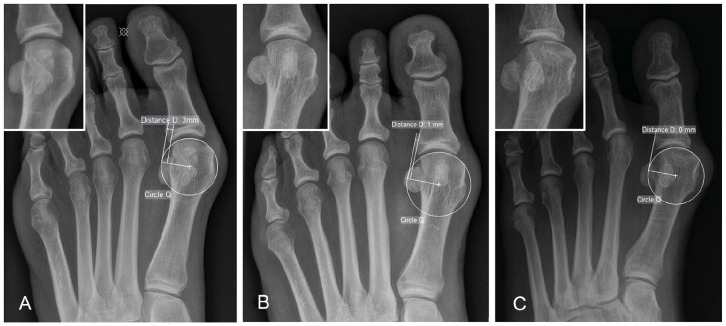
Dorsoplantar weightbearing foot radiographs with assessment of the round sign. A circle Q was drawn to the articular surface of the metatarsal head based on 3 points of contact (medial edge, lateral edge, and top of the head). (A) For the angular-shaped cases, the lateral cortical surface of the metatarsal head was not located on the circle Q, and the distance D to the circle Q was 2 mm or more. (B) For the intermediate cases, the distance was 1 mm. (C) For the round-shaped cases, the lateral cortical surface was situated on the circle Q.

Hallux valgus recurrence was defined as an HVA ≥20 degrees at the most recent follow-up.^2^ The HVA, IMA, DMAA, proximal to distal articular angle, and the first MTPJ congruency angle were measured as described previously.^3,12^ The sesamoid position was graded by the Hardy and Clapham classification.^10^ A grade of 5 or higher was defined as a lateral displacement of the tibial sesamoid.^20^ First metatarsal pronation was indirectly determined by the shape of the lateral edge of the first metatarsal head. According to Okuda’s^21^ circle method, the lateral edge was categorized as angular, intermediate, or round (Figure 2). The round sign was defined as positive if the shape was round and negative if it was angular or intermediate.^21^ Metatarsus adductus was defined by a metatarsus adductus angle of more than 20 degrees using the modified Sgarlato method.^1^ Flatfoot deformity was defined by a Meary angle of fewer than –4 degrees on the lateral radiographs, measuring the angle between the longitudinal axis of the first metatarsal and the talus.^28^

### Statistical Analysis

All radiologic parameters were tested for normality with the Shapiro Wilk test. As the data showed no normal distribution, the values were presented as medians and interquartile ranges (IQRs). The Wilcoxon signed-rank test was used to compare changes in the variables between the follow-ups. The radiologic variables of all cases were grouped according to recurrence and nonrecurrence. The Mann-Whitney *U* test was performed to reveal differences between the groups. Nominal or ordinal variables were expressed as numbers and percentages. Categorical variables were analyzed using the χ^2^ test. The McNemar test was performed to investigate the changes in the round sign between the follow-ups. Univariate analyses were used to search for risk factors for hallux valgus recurrence. Multivariate logistic regression analyses were performed including preoperative and postoperative variables with *P* values <.05 from the univariate analyses. Receiver operating characteristic (ROC) curve analyses determined cutoff values for the significant radiologic values. The variables were dichotomized for the logistic regression analysis according to the cutoff values. The results were reported as odds ratios (OR) with 95% CIs. All data were assessed using SPSS, version 28.0 (IBM Corp, Armonk, NY). In general, differences with a *P* <.05 were considered statistically significant.

## Results

After application of the exclusion criteria, 266 cases (240 females, 26 males) with a median age of 45.5 (range, 19-80) years were available for radiologic assessment. An additional Akin (closed-wedge osteotomy of the proximal phalanx) was performed in 110 cases (41.4%). Hallux valgus recurrence was observed in 23 of 266 cases (8.6%). There was no significant difference between the recurrence and nonrecurrence group with regard to age (*P* = .65), gender (*P* = .86), follow-up (*P* = .61), and additional Akin osteotomy (*P* = .52).

All radiographic measurements were conducted based on radiographs performed at an early follow-up (median, 6.2 weeks; range 5.8-10.3) and the most recent follow-up (median, 13 months; range, 12-177). The HVA, IMA, DMAA, proximal to distal articular angle, and the first MTPJ congruence angle were significantly reduced from preoperatively to the early follow-up (*P* < .001 for all measured values; Table 1). Preoperatively, the lateral edge of the metatarsal head was round in 40.2%, intermediate in 27.4%, and angular in 32.3% of the cases.

**Table 1.** Radiologic Parameters Measured Preoperatively, at the Early Follow-up, and at the Most Recent Follow-up.^a^

Parameters	Preoperative	Early Follow-up	Most Recent Follow-up
HVA	25.4 (21.1-30.5)	10.0 (6.5-14.0)	11.0 (9.3-15.8)
IMA	10.9 (9.3-12.9)	2.8 (2.8-6.2)	5.6 (3.5-7.2)
DMAA	11.1 (7.1-14.3)	6.1 (2.7-10.3)	6.0 (2.6-9.8)
PDPAA	7.7 (5.0-10.6)	6.2 (0.8-10.4)	5.4 (1.0-10.1)
First MTPJ incongruency angle	10.8 (6.1-17.0)	0.3 (−3.8 to 4.7)	1.4 (−3.3 to 5.9)
Round sign			
Angular	86 (32.3)	173 (65.0)	158 (59.4)
Intermediate	73 (27.4)	49 (18.4)	55 (20.7)
Round	107 (40.2)	44 (16.5)	53 (19.9)
Sesamoid position			
1	2 (0.8)	145 (54.5)	73 (27.4)
2	9 (3.4)	63 (23.7)	50 (18.8)
3	61 (22.9)	48 (18.0)	103 (38.7)
4	66 (24.8)	9 (3.4)	32 (12.0)
5	84 (31.6)	1 (0.4)	6 (2.3)
6	26 (9.8)	0	2 (0.8)
7	18 (6.8)	0	0

Abbreviations: DMAA, distal metatarsal articular angle; HVA, hallux valgus angle; IMA, intermetatarsal angle; MTPJ, metatarsophalangeal joint; PDPAA, proximal to distal phalangeal articular angle.

a Data presented as median (interquartile range) or as n (%).

**Figure 3.**
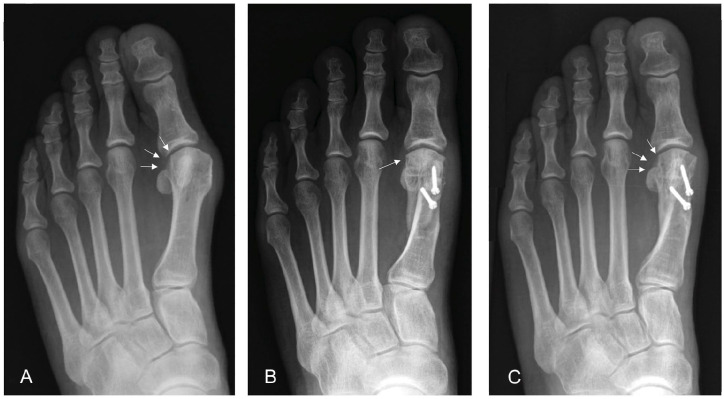
Dorsoplantar weightbearing foot radiographs showing the changes in the round sign over time. The preoperatively round-shaped lateral metatarsal head (A, arrows) changed to an angular shape at the 6-week follow-up (B, arrowhead) and changed back to a round shape until at the 1-year follow-up (C, arrows).

The univariate analyses revealed that a higher preoperative HVA, a positive round sign, and metatarsus adductus were significantly associated with hallux valgus recurrence (Table 2). Logistic regression analysis confirmed an HVA >30 degrees (OR 5.3, 95% CI 2.1, 13.7; *P* < .001), a positive round sign (OR 3.3, 95% CI 1.2, 8.7; *P* = .02), and metatarsus adductus (OR 4.0, 95% CI 1.6, 10.2; *P* = .004) as independent risk factors for hallux valgus recurrence. At the early follow-up, 63 of the 107 cases (58.9%) with a preoperatively round-shaped metatarsal had changed to an intermediate or an angular shape (*P* < .001), whereas the changes from the early to the most recent follow-up were not significant (*P* = .08; Table 3 and Figure 3). Still, a postoperative positive round sign was significantly associated with recurrence (*P* < .001; Table 4). The logistic regression analysis found a postoperative HVA >15 degrees (OR 70.0, 95% CI 13.8, 354.5; *P* < .001) and a postoperative positive round sign (OR 6.2, 95% CI 1.7, 22.2; *P* = .005) as independent risk factors for hallux valgus recurrence (Supplemental Table S1). Hallux valgus recurrence was present in 12 of the 53 cases (22.6%) with a positive round sign, whereas only 11 of the 213 cases (5.2%) with a negative round sign showed recurrence at the most recent follow-up.

**Table 2.** Univariate Radiologic Risk Factor Analysis of Preoperative Radiographic Parameters for Hallux Valgus Recurrence After ReveL Osteotomy.^a^

Preoperative Parameters	Hallux Recurrence (n = 23; 8.6%)	No Recurrence (n = 243; 91.4%)	Unadjusted *P* Value*
HVA	32.4 (28.6-34.9)	24.8 (20.6-29.5)	**<.001**
IMA	12.5 (9.3-13.8)	10.8 (9.3-12.5)	.06
DMAA	14.5 (6.5-20.4)	11.0 (7.3-14.1)	.11
PDPAA	7.7 (3.3-12.0)	7.6 (5.0-10.6)	.38
MTPJ congruency angle	14.7 (6.5-24.7)	10.4 (6.1-16.4)	.49
Round sign			
Negative	7 (4.4%)	152 (95.6%)	
Positive	16 (15.0%)	91 (85.0%)	**.003**
Sesamoid position			
(<4)	3 (4.2%)	69 (95.8%)	
(≥4)	20 (10.3%)	174 (89.7%)	.11
Metatarsus adductus			
No	9 (4.7%)	181 (95.3%)	
Yes	14 (18.4%)	62 (81.6%)	**<.001**
Flat foot deformity			
No	11 (6.6%)	156 (93.4%)	
Yes	12 (12.1%)	87 (87.9%)	.12

Abbreviations: DMAA, distal metatarsal articular angle; HVA, hallux valgus angle; IMA, intermetatarsal angle; MTPJ, metatarsophalangeal joint; PDPAA, proximal to distal phalangeal articular angle.

a Data presented as median (interquartile range) or as n (%).

* *P* value <.05 was set as statistically significant.

**Table 3.** Changes of the Round Sign Over Time.

Round Sign	Negative, n (%)	Positive, n (%)	*P* Value*
Preoperative	159 (59.8)	107 (40.2)	
Early follow-up	222 (83.5)	44 (16.5)	**<.001**
Most recent follow-up	213 (80.1)	53 (19.9)	.08

* *P* value <.05 was set as statistically significant.

**Table 4.** Univariate Radiologic Risk Factor Analysis of Early Postoperative Radiographic Parameters for Hallux Valgus Recurrence After ReveL Osteotomy.^a^

Postoperative Parameters	Hallux Recurrence (n = 23; 8.6%)	No Recurrence (n = 243; 91.4%)	Unadjusted *P* Value*
HVA	19.2 (16.5-21.6)	9.6 (6.2-12.9)	**<.001**
IMA	5.6 (3.7-6.9)	4.3 (2.8-6.0)	**.049**
DMAA	9.3 (5.0-14.4)	5.9 (2.5-10.3)	**.014**
PDPAA	6.6 (–5.2 to 11.3)	6.1 (1.2-10.4)	.69
First MTPJ incongruency angle	6.4 (–1.1 to 14.0)	0.1 (–4.1 to 4.0)	**.001**
Round sign			
Negative	13 (5.9)	209 (94.1)	
Positive	10 (22.7)	34 (77.3)	**<.001**
Sesamoid position			
<4	19 (7.4)	237 (92.6)	
≥4	4 (40.0)	6 (60.0)	**<.001**

Abbreviations: DMAA, distal metatarsal articular angle; HVA, hallux valgus angle; IMA, intermetatarsal angle; MTPJ, metatarsophalangeal joint; PDPAA, proximal to distal phalangeal articular angle.

a Data presented as median (interquartile range) or as n (%).

* *P* value <.05 was set as statistically significant.

## Discussion

The current study investigated the derotational effect of the distal translational ReveL osteotomy. The most important finding is that the first metatarsal round sign changed from positive to negative in 59% of the cases, partially confirming the study’s primary hypothesis. Because the ReveL osteotomy has no or only minimal corrective power of first metatarsal derotation in the coronal plane, we can only explain these round sign changes as a result of rebalancing of the metatarsal-sesamoid complex. Mortier et al^19^ suggested that the metatarsal-sesamoid ligaments act as a “drive belt” that forces the first metatarsal and phalanx into pronation as the medial capsule loosens and the intermetatarsal ligament remains tight, which finally results in a dislocation of the sesamoids from the metatarsal-sesamoid facets. The ReveL osteotomy, combined with the release of the metatarsal-sesamoid ligament and joint capsule, shifts the metatarsal head fragment laterally and reduces the fragment underneath the sesamoids. This reduction may reverse the pronation, which consequently may result in the round sign turning negative. Our results are in accordance with the findings of a previous study that investigated the derotational effect of 30 scarf osteotomies with a transarticular lateral release. The authors also found a significant reduction in the round sign prevalence from 40% to 13%.^24^

Based on this study’s results, it remains unclear why the remaining 41% of round-shaped cases could not be changed. The etiology and the exact location of first ray pronation are still debated. Therefore, metatarsal pronation may be due to ligamentous laxity, causing rotation in the joints of the first ray, an intrinsic rotation of the bones themselves, or a combination of both. In a computed-tomographic study, Kimura et al^15^ found the medial cuneiform to be significantly more pronated relative to the navicular, whereas the first metatarsal was supinated to the medial cuneiform. Others found intrinsic pronation within the first metatarsal bone itself.^4,26^ Besides the incomplete reduction of the metatarsal-sesamoid complex, these anatomical variations could possibly explain why metatarsal pronation could not be reversed in all cases.

Hallux valgus recurrence was significantly higher in cases with a preoperative positive round sign (15.0% vs 4.4%; *P* = .003), which confirms the study’s secondary hypothesis and further supports the theory that metatarsal pronation plays a major role not only in hallux valgus development but also in hallux valgus recurrence. A persistent positive round sign at the early follow-up increased the risk for recurrence even more (22.7% vs. 5.9%; *P* < .001) and was an independent risk factor in the logistic regression analysis (OR 6.2, *P* = .005). Park et al^23^ performed a risk factor analysis on hallux valgus recurrence in 131 feet that underwent proximal chevron osteotomy with distal lateral MTPJ release, defining an HVA ≥20 degrees as recurrence. They found a higher preoperative HVA ≥40 degrees and metatarsus adductus ≥23 degrees to be independent risk factors for recurrence, which is similar to the findings in our study. In contrast to our study, they did not find a postoperative round sign to be correlated with recurrence. This discrepancy may be explained by the fact that Park’s postoperative evaluation was based on nonweightbearing radiographs. Although we routinely perform intraoperative fluoroscopy to document the immediate postoperative correction, we consciously decided against evaluating this data, as it was performed in a nonstandardized manner and without full weightbearing.

The goal to reduce metatarsal pronation caused a trend toward proximal derotational metatarsal osteotomies and first metatarsal-cuneiform joint fusions.^5,16,29,31^ Yasuda et al^31^ performed a proximal supination osteotomy in 66 feet. The prevalence of a positive round sign significantly decreased from preoperative to postoperative (80% vs 20%; *P* < .0001). At a mean follow-up of 34 months, the overall recurrence rate was 4%, lower than in our study cohort. However, they also used a higher cutoff for recurrence (HVA > 25 degrees).^31^ Wagner and Wagner^29^ investigated 25 cases that underwent proximal rotational metatarsal osteotomy at the 1-year follow-up. There was no recurrence (HVA increase >10 degrees from immediate to final follow-up or HVA >15 degrees at final follow-up). The round sign was corrected in 24 of 25 cases.^29^ Dayton et al^5^ found only 1 recurrence in 109 feet that received a triplanar tarsometatarsal arthrodesis at a mean follow-up of 17 months. They attributed this low recurrence rate to the fact that all 93 cases with a positive round sign were corrected because of intentional supination of the first metatarsal during the procedure.^5^ Direct derotation of the first metatarsal may correct metatarsal pronation more accurately compared with the ReveL technique, which only allows indirect derotation by rebalancing of the soft tissues. However, it seems that not all hallux valgus deformities may need direct derotation to correct metatarsal pronation. Furthermore, the superiority of these direct derotational techniques has still to be proven as prior studies were limited by either small sample sizes or a short-term follow-up.

Limitations of the study were the retrospective design with a lack of axial radiographs or weightbearing CT scans for the staging of the sesamoids and direct measurement of metatarsal pronation. In the present study, we used the presence of a round sign as an indicator of first metatarsal pronation. The presence of a round shape or an intermediate shape may indicate degrees of metatarsal pronation, but without 3D weightbearing imaging we cannot confirm that. In theory, the Hardy and Clapham method indirectly classifies the position of the medial sesamoid but does not show the actual position of the sesamoid within the articular grooves of the metatarsal head. However, Kim et al^14^ demonstrated that the sesamoid position on simple radiographs does not correlate with the true subluxation of sesamoids. Yamaguchi et al^30^ showed that the round sign is significantly associated with metatarsal pronation and is turned negative in most cases as the pronation angles decreased from 10 to 0 degrees. Nevertheless, a recent study questioned the reliability of the round sign in predicting metatarsal pronation.^18^ The authors found a low correlation (*R*^2^: 0.15) between the round sign and first metatarsal pronation as measured on weightbearing CT scans. They explained their findings by categorization errors of the round sign because of the superposition of the sesamoids with the lateral edge of the metatarsal head and the presence of first MTPJ arthritis. To minimize the influence of this potential source of error, we excluded all patients who underwent additional cheilectomy for concomitant first MTPJ arthritis. Furthermore, we paid special attention not to confuse the sesamoids with the metatarsal head by adjusting the contrast and brightness of the digital radiographs.

## Conclusion

The ReveL osteotomy resulted in a correction of the positive round sign in 59% of the cases, suggesting that not all hallux valgus deformities with metatarsal pronation may need a derotational bony correction to eliminate the round sign. However, it remains unclear why the remaining cases were not corrected. In this cohort, the round sign was found to be an independent risk factor for hallux valgus recurrence. Further prospective studies with 3-dimensional analyses of the entire first ray are necessary to better understand the effects and limitations of distal translational osteotomies on the correction of metatarsal pronation.

## Appendix

**Supplemental Table S1.** Multivariate Logistic Regression Analysis Estimating the Effect of Risk Factors for Hallux Valgus Recurrence Including All Early Postoperative Parameters From the Univariate Analyses With *P* < .05.

Postoperative Parameters	Odds Ratio (95% CI)	*P* Value*
HVA >15 degrees	70.0 (13.8, 354.5)	**<.001**
IMA >4 degrees	2.3 (0.6, 8.2)	.20
DMAA >8 degrees	1.0 (0.3, 3.1)	.96
MTPJ congruency angle >0°	0.6 (0.2, 2.4)	.50
Positive round sign	6.2 (1.7, 22.2)	**.005**
Sesamoid position ≥4	2.9 (0.5, 15.6)	.22

Abbreviations: CI, confidence interval; DMAA, distal metatarsal articular angle; HVA, hallux valgus angle; IMA, intermetatarsal angle; MTPJ, metatarsophalangeal joint.

* *P* value <.05 was set as statistically significant.
